# Non-inferiority and Safety of Increased Povidone-Iodine (Betadine) Concentration for Irrigation Following Primary Total Joint Arthroplasty (TJA)

**DOI:** 10.7759/cureus.53453

**Published:** 2024-02-02

**Authors:** Yoav S Zvi, Lisa Y Follett, Hemant Reddy, Zeynep Seref-Ferlengez, Mitchell C Weiser, Eli Kamara

**Affiliations:** 1 Orthopedic Surgery, Montefiore Medical Center, Bronx, USA

**Keywords:** povidone-iodine, infection, irrigation, betadine, total joint arthroplasty

## Abstract

Introduction

Diluted Betadine (Purdue Pharma, Stamford, Conn) irrigation following primary total joint arthroplasty (pTJA) may reduce the risk of periprosthetic joint infection (PJI). A recent *in vitro* study found a minimal inhibitory concentration (MIC) of 0.63% Povidone-iodine (Betadine) for several bacterial isolates. This study reports outcomes of patients undergoing TJA using 0.54% Betadine irrigation compared to a historical cohort using 0.3% Betadine irrigation.

Methods

A retrospective chart review of patients who underwent pTJA from September 2017 to December 2020. 0.3% Betadine was used in a historical cohort and 0.54% Betadine in the experimental group. Patient demographics, intra-operative data, all-cause revision, and infection data were collected for the three-month post-operative period. Outcome frequencies between groups were compared using Fisher-Exact tests.

Results

Six hundred sixty-one patients underwent pTJA: 308 total knee arthroplasty (TKA), and 353 total hip arthroplasty (THA). 0.3% Betadine group had seven (3.1%) revisions: five (2.2%) underwent a revision for non-infectious reasons, and two (0.9%) for PJI. 0.54% Betadine group had 11 (2.5%) revisions: nine (2.1%) underwent revision for non-infectious reasons, two (0.4%) for PJI. No significant difference was found for rates of all-cause revision or infection between groups. No adverse intra-operative events occurred with the higher Betadine concentration.

Conclusion

This study demonstrated no difference in rates of all-cause revision or PJI when using 0.3% Betadine versus 0.54% Betadine for irrigation following pTJA. No adverse intraoperative events occurred with 0.54% Betadine irrigation. Given recent *in vitro *data supporting increased Betadine MIC, our results showed safety and non-inferiority with respect to three-month post-operative complication rates. Further investigation through a large powered randomized controlled study is needed to determine the optimal Betadine irrigation concentration for PJI prevention is required.

## Introduction

Periprosthetic joint infection (PJI) is a rare but devastating post-operative complication following primary total joint arthroplasty (pTJA) that can lead to significant patient morbidity and healthcare costs [1]. Surgeons utilize several techniques to mitigate the risk of post-operative PJI including administration of pre-operative antibiotics, sterile draping, and intra-operative wound irrigation. A variety of different solutions such as antibiotics, antiseptics and soaps have been utilized for wound irrigation in an effort to minimize rates of infection [2]. Povidone-iodine (Betadine) is an inexpensive, antiseptic solution that has been widely used for intra-operative wound irrigation due to its proven bactericidal properties against various pathogens and efficacy in preventing surgical site infection (SSI) [3,4]. A previous study reported on the use of a dilute Betadine concentration of 0.35% for intra-operative wound irrigation following final prosthetic implantation [5]. However, the optimal concentration and duration of wound irrigation is still up for debate. More recently, an in vitro study found a minimal inhibitory concentration (MIC) of 0.63% Betadine for several bacterial isolates [6]. In light of these findings, we performed a case series study at our institution to report on outcomes of patients undergoing pTJA using 0.54% Betadine irrigation, compared to a historical cohort undergoing pTJA using 0.3% Betadine irrigation.

## Materials and methods

Study population

Approval from the local investigational review board was obtained prior to the initiation of this study. We performed a retrospective chart review using an institutional database of all patients who underwent unilateral primary total knee arthroplasty (TKA) or total hip arthroplasty (THA) at a single academic medical center in the Bronx, NY, between the months of September 2017 and December 2020. Surgeries were performed by two fellowship-trained board-certified orthopedic arthroplasty surgeons at the same academic institution.

Inclusion criteria

Patients with Current Procedural Terminology (CPT) code 27447 for a unilateral pTKA and CPT code 27130 for a unilateral pTHA were included in this study. Patients who were younger than 18 years of age at the time of surgery and patients who were admitted for reasons other than pTJA and underwent pTJA were excluded (i.e., sarcoma resection and TKA).

Patient demographics

An administrative database was created and used to collect patient demographic information including age, gender, race/ethnicity, American Society of Anesthesiologists physical health score (ASA status), insurance type, smoking status, and ICD-10 codes for several medical co-morbidities.

Outcome measures

The primary outcomes in this study were all post-operative complications occurring during the three-month post-operative period. These complications included manipulation, return to OR, closed reduction, revision arthroplasty for non-infectious reasons (i.e., aseptic loosening, instability, fracture, etc.), revision wound closure with irrigation and debridement (I&D) +/- head/liner or liner exchange for non-infectious reasons (wound drainage, wound dehiscence, skin necrosis, scar revision, seroma, etc.), and revision arthroplasty for deep infectious reasons (i.e. I&D with head/liner or liner exchange, two-stage revision w/ antibiotic spacer). Additional data regarding intra-operative adverse events (i.e. anaphylaxis) were collected as well; however, no patients demonstrated any such findings.

Pre-operative antibiotic prophylaxis, dilute Betadine preparation, and intra-operative use

Patients were administered intravenous cefazolin at the standard weight-dependent dosage within 60 minutes prior to surgical incision. Patients with severe allergy to cefazolin were administered either clindamycin or vancomycin. To prepare the dilute Betadine irrigation, a sterile povidone-iodine ophthalmic solution was used (Betadine 5%, Alcon Laboratories, Fort Worth, Texas). The intra-operative wound irrigation for the historical patient cohort using 0.3% Betadine irrigation was prepared by mixing 30cc of 5% Betadine with 470cc of normal saline. The 0.54% Betadine wound irrigation was prepared by mixing 30cc of 5% Betadine with 250cc of normal saline. Although previously mentioned in vitro studies have shown an MIC of 0.63% Betadine to be effective against the most commonly isolated pathogens in PJI, 0.54% concentration was chosen as a dilution of convenience, given the ease of titrating to this level. Both surgeons performed their intra-operative wound irrigation following final implantation and prior to skin closure. The surgical site was exposed to the dilute Betadine irrigation for approximately three minutes, which was timed by the surgical team using a standard analog clock. The wounds were then thoroughly irrigated with a 0.9% sodium chloride solution by pulse lavage to remove excess dilute Betadine irrigation prior to wound closure. 

Statistical analysis

Continuous variables are reported as mean (standard deviation) and categorical variables are reported as frequencies (percentages, %). Patient baseline characteristics and outcome frequencies between the two groups were compared using Chi-square and Mann-Whitney U tests for categorical and continuous variables, respectively.

## Results

A total of 661 patients included in this study underwent pTJA. There were 227 patients in the historical cohort using 0.3% Betadine irrigation (104 pTKA and 123 pTHA), and 434 patients in the experimental cohort using 0.54% Betadine irrigation (204 pTKA and 230 pTHA). To control for possible confounding variables with respect to patient characteristics, we compared patient age, BMI, gender, ASA score, and smoking status between the two groups. No significant differences were detected in any of these variables between groups (Table 1). The 0.3% Betadine group had two (1.6%) patients requiring post-operative manipulation, while the 0.54% Betadine group also had two (0.9%) patients requiring manipulation; there was no statistical significance between the two groups. Only one (0.2%) patient in the 0.54% Betadine group required a closed reduction. The 0.3% Betadine group had a total of seven (3.1%) patients return to the OR. Of those, one (0.4%) patient required revision for aseptic loosening, and four (1.8%) underwent revision wound closure for non-infectious reasons. Deep infection requiring I&D and head/liner or liner exchange occurred in two (0.9%) patients, while no patients developed deep infection requiring two-stage revision with an antibiotic spacer. The 0.54% Betadine group had a total of 11 (2.5%) patients return to the OR. Of those, three (0.7%) patients underwent revision arthroplasty for non-infectious reasons, and five (1.2%) required revision wound closure with I&D and head/liner or liner exchange for non-infectious reasons. Deep infection requiring I&D and head/liner or liner exchange occurred in one (0.2%) patient, while one (0.2%) patient developed a deep infection requiring two-stage revision with an antibiotic spacer. No significant difference was found for rates of return to OR, closed reduction, revision, or infection between the two groups. No adverse intra-operative events occurred with higher Betadine concentration. These results are summarized in Table 2.

**Table 1:** Comparison of demographic and medical characteristics between both groups of patients

	0.3% Betadine	0.54% Betadine	P-value
Age (mean ± SD)	62.9 ± 11	64.0 ± 9	0.6
BMI (mean ± SD)	31.0 ± 6.8	31.2 ± 6.6	0.6
Gender			0.1
Female	149 (66%)	286 (66%)	
Male	78 (34%)	140 (33%)	
ASA Score			0.2
1-2	105 (46%)	189 (44%)	
3-4	122 (54%)	237 (55%)	
Smoke	34 (15%)	82 (19%)	0.2

**Table 2:** Comparison of post-operative complications and return to OR rates between both groups of patients

	0.3% Betadine	0.54% Betadine	P-value
Total Cases	227	434	
Total Knee Arthroplasty	104	204	
Total Hip Arthroplasty	123	230	
Manipulation	2 (1.6%)	2 ( 0.9%)	NS
Return to OR	7 (3.1%)	11 (2.5%)	NS
Closed Reduction	0 (0%)	1 (0.2%)	NS
Revision - No Infection (instability, fracture, etc.)	1 (0.4%)	3 (0.7%)	NS
Revision Closure, I&D +/- head/liner or liner exchange (Drainage, Wound dehiscence, skin necrosis, scar revision, seroma)	4 (1.8%)	5 (1.2%)	NS
Deep-Infection I&D, Liner Exchange	2 (0.9%)	1 (0.2%)	NS
Deep-Infection 2-Stage Revision	0 (0%)	1 (0.2%)	NS

## Discussion

In this study, we found that using 0.54% Betadine for intra-operative irrigation following pTJA was non-inferior to 0.3% Betadine when comparing rates of post-operative PJI. No significant differences were observed in all-cause revision or PJI rates between the two groups. It is important to note that our study is significantly underpowered and would require a sample size of 14,209 participants per group in order to detect a meaningful difference. While our findings suggest safety and noninferiority, a larger, more thorough study is needed to determine the optimal Betadine irrigation concentration for preventing PJI in pTJA.

With the increasing number of pTJA procedures performed nationwide, the rates of revision arthroplasty for PJI are increasing as well [7]. In addition to the significant patient morbidity associated with these procedures, costs for revision arthroplasty for PJI have been rapidly increasing - more costly than pTJA and nearly double the costs for aseptic revision TJA [8,9]. The significant morbidity and healthcare costs associated with PJIs have driven the orthopedic community to investigate the efficacy of various antibacterial and antiseptic agents in an effort to minimize intra-operative contamination and bioburden from varying pathogens [10,11].

Several studies have demonstrated dilute Betadine to be a safe and inexpensive antiseptic solution that can reduce rates of surgical site infection (SSIs) in multiple medical fields. Chundamala et al. performed a systematic review to determine the efficacy and risks of using Betadine irrigation to prevent SSIs; their study included 11 randomized controlled trials and four prospective comparative studies in the general surgery, urologic, spine, and orthopedic medical fields [4]. They concluded that Betadine was significantly more effective in preventing SSI when compared to saline, water, or no irrigation. In addition, they found no significant risks in the use of Betadine irrigation aside from increased post-operative serum iodine. In the orthopedic spine literature, Cheng et al. performed a prospective, single-blinded randomized study to evaluate the efficacy of dilute Betadine on rates of wound infection after spinal surgery [12]. They found that patients undergoing intra-operative wound irrigation with saline water alone had significantly increased rates of deep and total infection rates when compared to patients undergoing intra-operative wound irrigation with 0.35% Betadine. Brown et al. evaluated the effect of dilute Betadine irrigation on PJI following pTJA [5]. Their protocol for intra-operative irrigation involved 0.35% Betadine lavage for 3 minutes and compared this to a historical cohort using only saline irrigation. They found a total of 18 early post-operative infections prior to the adoption of their dilute Betadine irrigation protocol, which was significantly higher than their cohort undergoing dilute Betadine irrigation.

There is ample literature demonstrating the antiseptic properties of Betadine as well as its efficacy in minimizing the risk of PJI. This has led the World Health Organization to advocate for the use of dilute Betadine for wound irrigation during surgical procedures [13]. Moreover, a recent international consensus statement on orthopedic infections received a “strong” recommendation in favor of diluting Betadine irrigation during clean elective orthopedic surgeries [14]. There are currently no specific guidelines on the optimal concentration or wound exposure time when using dilute Betadine. More recent in vitro data has demonstrated a maximum MIC of 0.63% for Betadine to be effective against bacterial isolates commonly implicated in PJI; this included methicillin-resistant Staphylococcus aureus (MRSA) (0.08%), Haemophilus influenzae (0.16%), pseudomonas aeruginosa (0.31%), Burkholderia cepacia (0.63%), escherichia coli (0.63%), staphylococcus epidermidis strain A (0.16%) and staphylococcus epidermidis strain B (0.16%) [6]. The findings of this study served as the rationale behind the use of an increased Betadine concentration at our institution. Additionally, Betadine is extremely cost-effective. Other commercial PJI antiseptic solutions may cost up to $300 per usage, while the Betadine used at our institution is $20 per vial, and a single vial is used per case.

Our study had a number of limitations that should be mentioned. First, this was a retrospective chart review using a historical cohort that may not be representative of the general population. Furthermore, there may be inherent temporal bias in the patient outcomes given the fact that both surgeons utilized 0.3% Betadine irrigation earlier in their careers and then later switched to 0.5% irrigation. This may introduce bias with respect to surgical abilities as they may have improved over time and helped lower complication rates. Lastly, our study is significantly underpowered. A retrospective power analysis was calculated and revealed that in order to achieve a satisfactory level of statistical power, a study population of 14,209 participants in each experimental group would be required. This step is critical as it decreases the likelihood of type 2 error and increases the reliability of findings.

This study reported on the safety and efficacy profile of an increased 0.54% Betadine irrigation solution, as compared to a historical cohort of 0.3% Betadine irrigation solution. We demonstrated no difference in rates of return to OR, revision, or PJI when using 0.3% Betadine versus 0.54% Betadine for irrigation following pTJA. No adverse intraoperative events occurred with 0.54% Betadine irrigation. Given recent in vitro data showing increased Betadine MIC, our results showed safety and non-inferiority with respect to three-month post-operative complication rates. Further investigation to determine optimal Betadine concentrations for irrigation following pTJA to minimize the risk of PJI is required.

## Conclusions

This study demonstrated no difference in rates of all-cause revision or PJI when using 0.3% Betadine versus 0.54% Betadine for irrigation following pTJA. No adverse intraoperative events occurred with 0.54% Betadine irrigation. Given recent in vitro data supporting increased Betadine MIC, our results showed safety and non-inferiority with respect to three-month post-operative complication rates. Further investigation to determine the optimal Betadine irrigation concentration for PJI prevention is required.
